# Enhanced circulating transforming growth factor beta 1 is causally associated with an increased risk of hepatocellular carcinoma: a mendelian randomization meta-analysis

**DOI:** 10.18632/oncotarget.13218

**Published:** 2016-11-08

**Authors:** Wei-Qun LU, Ji-Liang QIU, Zhi-Liang HUANG, Hai-Ying LIU

**Affiliations:** ^1^ Department of Gastrointestinal Tumor Surgery, Cancer Center of Guangzhou Medical University, Guangzhou, Guangdong, China

**Keywords:** hepatocellular carcinoma, transforming growth factor beta 1, polymorphism, meta-analysis, mendelian randomization

## Abstract

The aim of this study was to test the causal association between circulating transforming growth factor beta 1 (protein: TGF-β1 and coding gene: *TGFB1*) and hepatocellular carcinoma by choosing *TGFB1* gene C-509T polymorphism as an instrument in a Mendelian randomization (MR) meta-analysis. Ten English articles were identified for analysis. Two authors independently assessed each article and abstracted relevant data. Odds ratio (OR) and weighted mean difference (WMD) with 95% confidence interval (CI) were synthesized under a random-effects model. Overall, the association of C-509T polymorphism with hepatocellular carcinoma was negative, but its association with circulating TGF-β1 was statistically significant, with a higher concentration observed in carriers of the -509TT genotype (WMD, 95% CI, *P*: 1.72, 0.67–2.78, 0.001) and -509TT/-509TC genotypes (WMD, 95% CI, *P*: 0.98, 0.43–1.53, < 0.001). In subgroup analysis, C-509T polymorphism was significantly associated with hepatocellular carcinoma in population-based studies under homozygous-genotype (OR, 95% CI, *P*: 1.74, 1.08–2.80, 0.023) and dominant (OR, 95% CI, *P*: 1.48, 1.01–2.17, 0.047) models. Further MR analysis indicated that per unit increase in circulating TGF-β1 was significantly associated with a 38% (95% CI: 1.03–4.65) and 49% (95% CI: 1.01–6.06) increased risk of hepatocellular carcinoma under homozygous-genotype and dominant models, respectively. Conclusively, based on a MR meta-analysis, our findings suggest that enhanced circulating TGF-β1 is causally associated with an increased risk of hepatocellular carcinoma.

## INTRODUCTION

Transforming growth factor beta 1 (TGF-β1) is a polypeptide cytokine that belongs to the transforming growth factor beta super-family [1]. It is widely accepted that TGF-β1 acts as a crucial regulator of cell growth, proliferation, differentiation and apoptosis [2–4]. Epidemiologic studies have observed a significant higher concentration of circulating TGF-β1 in patients with cancer at many sites including the liver than in cancer-free controls [5–7]. It is hence reasonable to assume that elevated circulating TGF-β1 may be causally associated with an increased risk of developing hepatocellular carcinoma [8]. Determining the answer to this assumption is far from an easy proposition, but the introduction of Mendelian randomization (MR) may cast a new light on the cause-and-effect dissection using observational data.

In theory, MR means the usage of a genetic alteration as an instrument to dissect a causal effect of an intermediate phenotype on a disease and importantly this effect is not subject to reverse causation and confounding that often confuse the interpretation of observational findings [9, 10]. An immediate and direct approach for instrument selection revolves around the functional variation of encoded or relevant gene for the intermediate phenotype of interest [11, 12]. In humans, TGF-β1 is encoded by *TGFB1* gene (Gene ID: 7040) that is mapped on chromosome 19q13.1 and contains 7 exons. Experimental evidence lends support to the observation that *TGFB1* gene is frequently up-regulated in tumor cells and the loss of TGF-β signaling is proposed as a hallmark of carcinogenesis [13–15]. *TGFB1* gene is polymorphic in genomic sequences and it incorporates approximate 1700 validated bi-allelic polymorphisms. One of the most widely-evaluated polymorphisms is C-509T (rs1800469) in the promoter region of *TGFB1* gene and this polymorphism was reported to be associated with the significant changes of circulating TGF-β1 by many investigators [16, 17]. What's more, experimental evidence from reporter constructs suggested that expression of *TGFB1* gene differed significantly between the -509C allele and the -509T allele, and this difference may be caused by selective activator protein 1 (AP1) recruitment to its promoter [18]. To explore the possible causal association between circulating TGF-β1 and hepatocellular carcinoma, we thereby chose *TGFB1* gene C-509T polymorphism as an instrument and conducted a meta-analysis of available published studies under the principles of MR method.

## RESULTS

### Qualified studies

**Figure 1:**
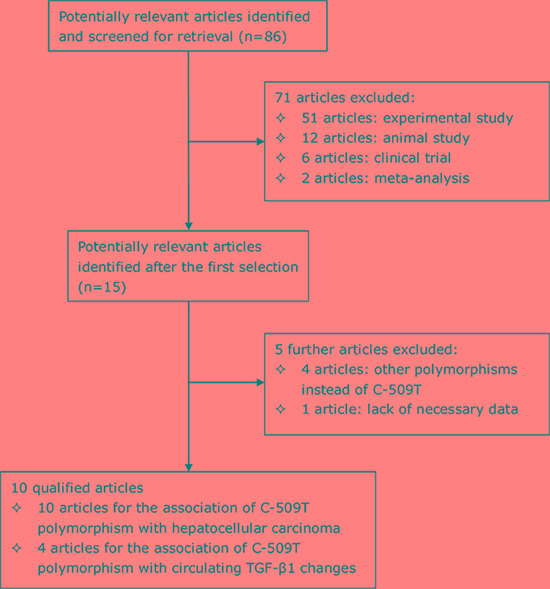
The selection process of qualified articles in this meta-analysis

Using the predefined inclusion criteria, 10 out of 86 retrieved articles were qualified for analysis [7, 16, 17, 19–25] and the selection process is schematized in Figure 1. As two articles from within included sub-studies according to the co-infection of hepatitis B and C viruses [16, 17], a total of 12 studies were qualified for the association of *TGFB1* gene C-509T polymorphism with hepatocellular carcinoma, involving 2809 patients with hepatocellular carcinoma and 4802 cancer-free controls. The basic characteristics of the 12 qualified studies are shown in Table 1. As for the association of C-509T polymorphism with circulating TGF-β1, there were 4 articles [7, 16, 17, 25] involving 10 independent comparisons and 1986 study subjects. The distributions of circulating TGF-β1 across C-509T genotypes are summarized in Table 2.

**Table 1:** The baseline characteristics of 12 qualified studies in this meta-analysis

Author (year)	Country	Source	Genotyping	Sample size		Age (yrs)		Males		HCV		HBV		Patients			Controls		
				Patients	Controls	Patients	Controls	Patients	Controls	Patients	Controls	Patients	Controls	-509TT	-509TC	-509CC	-509TT	-509TC	-509CC
Wan (2015)	S.China	Population	Chip	214	214	34.5	NA	0.72	NA	NA	NA	0.182	0.07	101	88	25	72	97	45
Ma (2015)	N.China	Population	RFLP	234	375	NA	NA	0.769	0.749	0	0	NA	NA	42	101	91	71	161	143
Ma (2015)	N.China	Population	RFLP	159	375	NA	NA	0.73	0.749	1	0	NA	NA	42	67	50	71	161	143
Yang (2012)	S.China	Hospital	Chip	772	852	NA	NA	0.807	0.779	NA	NA	0.786	0.306	109	360	303	118	384	350
Xin (2012)	N.China	Hospital	Chip	347	881	54.5	39.4	0.816	0.768	0	0	1	0	88	177	82	237	432	212
Shi (2012)	S.China	Hospital	RFLP	73	117	NA	NA	0.625	0.632	NA	NA	NA	NA	8	24	40	9	55	53
Radwan (2012)	Egypt	Population	RFLP	128	160	59.3	58.9	0.469	0.563	1	0	NA	NA	40	64	24	30	68	62
Miki (2011)	Japan	Hospital	Chip	212	765	66	65	0.764	0.531	1	1	0	0	59	107	46	194	379	192
Qi (2009)	S.China	Hospital	RFLP	379	299	58	55	0.654	0.719	0	0	1	0	92	198	89	93	156	50
Qi (2009)	S.China	Hospital	RFLP	379	196	55	56	0.654	0.73	0	0	1	1	92	198	89	64	101	31
Falleti (2008)	Italy	Population	RFLP	54	134	54	51	0.718	0.657	0.463	0.463	0.122	0.122	17	23	14	36	62	36
Kim (2003)	Korea	Hospital	Chip	237	809	55.6	49	0.814	0.739	0	0	1	1	18	134	76	99	487	187

***NOTE*:** S.China, south China; N.China, north China; HCV, hepatitis C virus; HBV, hepatitis B virus; RFLP, restricted fragment length polymorphism.

**Table 2:** The distributions of circulating TGF-β1 across *TGFB1* gene C-509T genotypes

Author (year)	Study subjects	Method for TGF-β1	-509TT			-509TC			-509CC		
			*N*	mean (ng/ml)	s.d. (ng/ml)	*N*	mean (ng/ml)	s.d. (ng/ml)	*N*	mean (ng/ml)	s.d. (ng/ml)
Wan (2015)	both HCC patients and controls	NA	173	39.45	7.45	185	26.33	13.65	70	26.25	13.56
Ma (2015)	controls	ELISA	71	4	1.5	161	3.6	1.2	143	3.3	1
Ma (2015)	HCC patients w/o HCV	ELISA	42	17.1	4.1	101	16.5	3.7	91	15.2	3.1
Ma (2015)	HCC patients w/h HCV	ELISA	42	23.1	4.4	67	21.8	3.7	50	20.3	3.3
Radwan (2012)	controls	ELISA	30	3.9	1.1	68	3.5	0.7	62	3.2	0.95
Radwan (2012)	cirrhosis patients with HCV	ELISA	44	15.5	2.8	74	14.6	2.1	34	13.4	2.6
Radwan (2012)	HCC patients with HCV	ELISA	40	20.5	3.3	64	19.5	1.8	24	18.3	2.2
Qi (2009)	controls	ELISA	42	10.13	4.19	54	9.64	4.51	24	10.43	5.54
Qi (2009)	HBV patients w/o HCC	ELISA	35	9.46	7.62	37	10.04	4.89	22	11.86	7.81
Qi (2009)	HCC patients w/t HBV	ELISA	36	8.98	5.8	74	9.75	6.36	26	12.83	8.72

***NOTE***: s.d., standard deviation; HCV, hepatitis C virus; HBV, hepatitis B virus; HCC, hepatocellular carcinoma; ELISA, enzyme-linked immunosorbent assay; NA, not available.

### Study characteristics

All qualified articles were published from the year 2003 to 2015 and total sample size ranged from 188 to 1624. Of 12 studies, 8 were performed in China (5 in southern China and 3 in northern China), 1 separately in Japan, South Korea, Italy and Egypt. Seven studies collected cancer-free controls from hospitals and five from general populations. The C-509T genotypes were determined by restricted fragment length polymorphism (RFLP) method in 7 studies and by chip-related methods in 5 studies. Eight studies had a total sample size of at least 500 and four studies of less than 500.

### C-509T polymorphism and hepatocellular carcinoma

**Figure 2:**
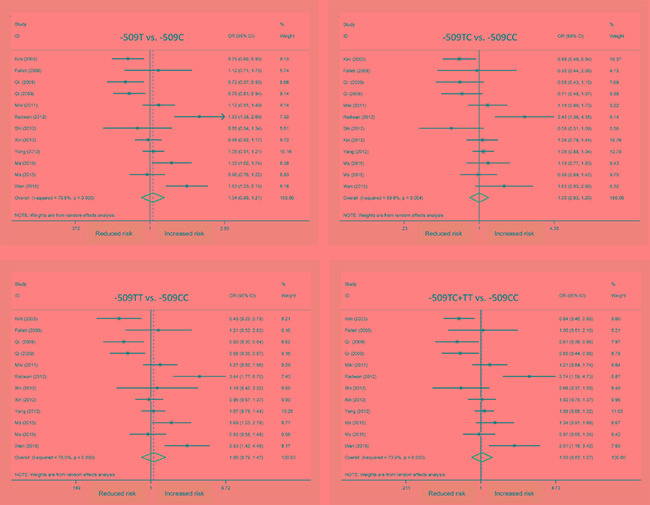
Forest plots of *TGFB1* gene C-509T polymorphism with hepatocellular carcinoma OR: odds ratio; 95% CI: 95% confidence interval. The x-axis represents the risk estimate OR.

**Figure 3:**
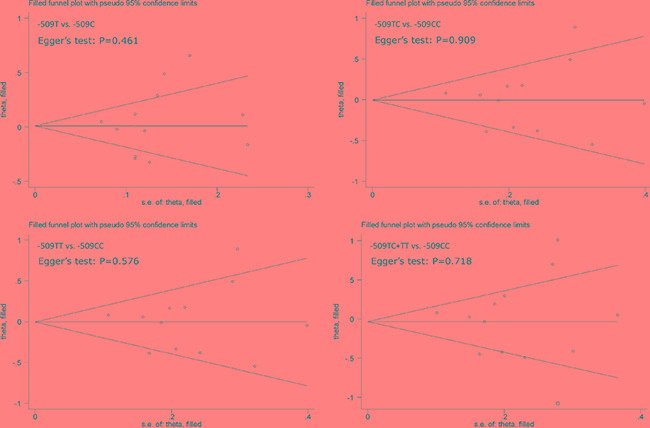
Funnel plots of *TGFB1* gene C-509T polymorphism with hepatocellular carcinoma

In overall analysis, C-509T polymorphism was not significantly associated with hepatocellular carcinoma under four genetic models (Figure 2) and this association was obsessed by significant heterogeneity (*I*^2^ > 50%). However, it was unlikely for the existence of publication bias as revealed by Egger's tests and filled funnel plots (Figure 3).

To identify possible sources of clinical heterogeneity, subgroups by country, source of controls, genotype method and sample size were conducted and effect-size estimates are presented in Table 3. Significant association was observed in studies with population-based controls under allelic (OR, 95% CI, *P*: 1.35, 1.05–1.74, 0.021), homozygous-genotype (OR, 95% CI, *P*: 1.74, 1.08–2.80, 0.023) and dominant (OR, 95% CI, *P*: 1.48, 1.01–2.17, 0.047) models, with significant statistical heterogeneity. Additionally, in studies with a total sample size of less than 500, carriers of the -509TT genotype vis-à-vis the -509CC genotype were 2.07-times more likely to develop hepatocellular carcinoma (OR, 95% CI, *P*: 2.07; 1.26–3.41; 0.004) and heterogeneity was improved (*I*^2^ = 42.8%).

**Table 3:** Subgroup analysis of TGFB1 gene C-509T polymorphism with hepatocellular carcinoma

Group	Number of studies	T vs. C		TT vs. CC		TC vs. CC		TT+TC vs. CC	
		OR, 95% CI, *P*	*I^2^*	OR, 95% CI, *P*	*I^2^*	OR, 95% CI, *P*	*I^2^*	OR, 95% CI, *P*	*I^2^*
Sample size									
≥ 500	8	0.94, 0.82–1.08, 0.373	71.1%	0.86, 0.64–1.15, 0.311	72.0%	0.94, 0.80–1.10, 0.447	42.2%	0.92, 0.76–1.11, 0.382	64.3%
< 500	4	1.36, 0.97–1.91, 0.075	70.2%	2.07, 1.26–3.41, 0.004	42.8%	1.24, 0.66–2.33, 0.509	75.0%	1.42, 0.75–2.70, 0.282	78.8%
Country									
S.China	5	0.96, 0.73–1.25, 0.754	84.1%	0.95, 0.55–1.64, 0.857	82.7%	0.89, 0.65–1.22, 0.450	64.1%	0.90, 0.61–1.31, 0.567	77.7%
N.China	3	1.07, 0.88–1.28, 0.530	53.2%	1.12, 0.79–1.59, 0.535	49.1%	1.06, 0.87–1.31, 0.562	0.0%	1.07, 0.89–1.30, 0.475	0.0%
Source of controls									
Hospital	7	0.89, 0.78–1.02, 0.100	65.4%	0.79, 0.58–1.08, 0.135	67.8%	0.87, 0.71–1.06, 0.171	54.4%	0.84, 0.67–1.05, 0.119	65.4%
Population	5	1.35, 1.05–1.74, 0.021	72.5%	1.74, 1.08–2.80, 0.023	69.6%	1.33, 0.96–1.84, 0.092	50.2%	1.48, 1.01–2.17, 0.047	68.9%
Genotyping									
Chip	5	1.06, 0.87–1.29, 0.592	80.1%	1.08, 0.72–1.62, 0.722	78.5%	1.04, 0.83–1.31, 0.744	58.0%	1.07, 0.81–1.41, 0.646	74.4%
RFLP	7	1.03, 0.80–1.33, 0.824	81.8%	1.09, 0.65–1.80, 0.749	80.7%	0.96, 0.70–1.32, 0.786	65.3%	0.99, 0.69–1.43, 0.954	77.1%

***NOTE***: OR, odds ratio; 95% CI, 95% confidence interval; S.China, south China; N.China, north China; RFLP, restricted fragment length polymorphism.

After modeling age, gender and the percentages of hepatitis B and C virus infection in meta-regression analysis, we failed to detect any significance of these confounding factors under four genetic models (*P* > 0.05).

### C-509T polymorphism and circulating TGF-β1

**Figure 4:**
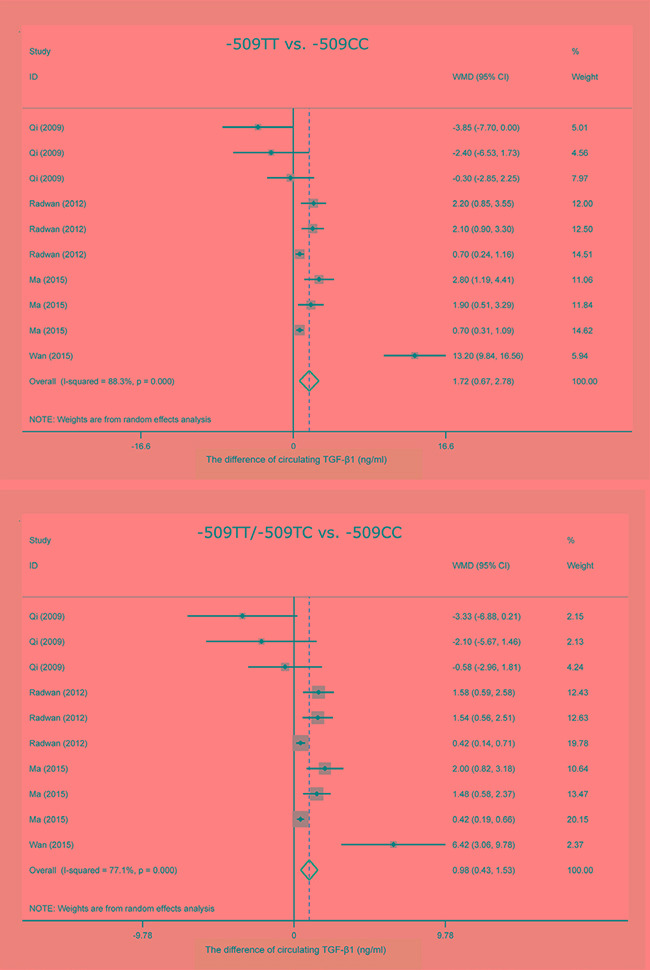
Forest plots of *TGFB1* gene C-509T polymorphism with circulating TGF-β1 changes WMD: weighted mean difference; 95% CI: 95% confidence interval.

Taking the -509CC genotype as a reference, carriers of the -509TT genotype (WMD, 95% CI, *P*: 1.72 ng/ml, 0.67–2.78 ng/ml, 0.001), the -509TC genotype (WMD, 95% CI, *P*: 0.59 ng/ml, 0.21–0.98 ng/ml, 0.003) and the -509TT/-509TC genotypes (WMD, 95% CI, *P*: 0.98 ng/ml, 0.43–1.53 ng/ml, < 0.001) had significant higher concentrations of circulating TGF-β1 in a random-effects model (Figure 4). However, heterogeneity was still an obsessing issue.

### MR analysis

In view of the prerequisites for MR, causal relevance was only estimated in studies with population-based controls under homozygous-genotype and dominant models. Per unit increase in circulating TGF-β1 was significantly and causally associated with a 38% (OR, 95 CI: 1.38, 1.03–4.65) and 49% (OR, 95 CI: 1.49, 1.01–6.06) increased risk of having hepatocellular carcinoma under homozygous-genotype and dominant models, respectively.

## DISCUSSION

This meta-analysis was designed to explore the causal association between circulating TGF-β1 and hepatocellular carcinoma by choosing *TGFB1* gene C-509T polymorphism as an instrument under the principles of MR method. Our findings provided evidence for the significant association of *TGFB1* gene C-509T polymorphism with hepatocellular carcinoma in population-based studies and circulating TGF-β1 changes, and importantly enhanced circulating TGF-β1 was suggested to be causally associated with an increased risk of hepatocellular carcinoma. To the best of our knowledge, this is the first report that has explored the cause-and-effect relationship between circulating TGF-β1 and hepatocellular carcinoma.

Hepatocellular carcinoma is the most common type of liver cancer and its incidence is soaring to endemic proportions, especially in Asian and African countries [26]. The incidence rate of hepatocellular carcinoma stems mainly from several risk factors, including gender, race/ethnicity, chronic viral hepatitis and alcoholism [27, 28]. There is also compelling evidence for genetic predisposition to hepatocellular carcinoma and *TGFB1* gene is one of promising candidates [29, 30]. Current literature is proliferating with a large volume of findings highlighting the susceptible role of *TGFB1* gene in hepatocellular carcinoma; however, there is little published knowledge revolving the genetic determinants of circulating TGF-β1 and their possible relationship with this disease. To fill this gap in knowledge, we introduced the concept of MR in this meta-analysis and produced convincing estimates for the possible causal involvement of circulating TGF-β1 in hepatocellular carcinogenesis by means of a well-defined promoter polymorphism in *TGFB1* gene as an instrument.

Although overall analysis failed to show any significance between *TGFB1* gene C-509T polymorphism and hepatocellular carcinoma, our subgroup analysis demonstrated that carriers of the -509TT genotype or the -509T allele vis-à-vis the -509CC genotype who had higher concentrations of circulating TGF-β1 were at a significantly increased risk in studies with population-based controls. Under the principles of MR, we propose for the first time the causal involvement of circulating TGF-β1 in the development of hepatocellular carcinoma. This involvement may be biologically plausible. Generally, there are two possible mechanisms for the involvement of TGF-β signaling in hepatocellular carcinoma, via intrinsically acting as an autocrine or paracrine growth factors and via extrinsically altering cancer-related microenvironments [31, 32]. A previous study has suggested that both circulating TGF-β1 and its mRNA levels can be used as sensitive biomarkers for the diagnosis and prognosis of hepatocellular carcinoma [33]. A more recent study further demonstrated that TGF-β1 expression was a reliable biomarker for predicting survival in patients with hepatocellular carcinoma after hepatic resection [34]. In addition, an animal study confirmed that activated TGF-β pathway underlay a novel tumor-promoting role of sulfatase 1 in hepatocellular carcinoma [35]. However, a key caveat should be raised as enhanced TGF-β1 in circulation might merely be a surrogate indicator of enhanced secretion of TGF-β1 by stromal cells via a paracrine activity, and the cross-talk between tumor cells and host stroma is reported to play a key role in carcinogenesis [31, 32]. There is indirect evidence supporting this claim that in patients with hepatocellular carcinoma, carriers of the -509CC genotype had statistically significant higher levels of circulating TGF-β1 and liver tumor tissue TGF-β1 mRNA expression compared with those with the -509TT genotype [17]. Exploring the correlation between circulating and stroma-secreted TGF-β1 is worthwhile, but is beyond the scope of this study. Nevertheless, on the basis of above independent lines of evidence and our current findings, circulating TGF-β1 can be exploited in clinical practice as a possible causal biomarker or a surrogate indicator of stroma-secreted TGF-β1 for the detection, diagnosis and prognosis of hepatocellular carcinoma.

Despite the promising results of this study, we must draw special attention to the MR method adopted in this meta-analysis. It is widely believed that testing for a cause-and-effect of a phenotype on a disease by testing an association between a genetic locus and the disease is reasonable and straightforward pending three key assumptions for a genetic locus quantified as an instrument variable, that is, the locus (i) should be robustly associated the phenotype, (ii) should not be associated with confounding factors that may bias the association between the phenotype and the disease and (iii) should exert its impact on the clinical outcome through the specific phenotype [36]. For the first assumption, the biological plausibility of *TGFB1* gene C-509T polymorphism is well defined, as this polymorphism is reported to account for a nearly twofold difference in circulating TGF-β1, which might be due to transcriptional suppression by the binding of activator protein 1 to this locus [18]. In addition, as this meta-analysis is not based on individual participant data and some necessary data are missing, it is beyond our ability to test the justification of the last two assumptions for the genetic instrument selected. Furthermore, the presence of linkage disequilibrium, genetic heterogeneity, pleiotropy, population stratification, canalization, or lack of knowledge about the confounding factors deserves special consideration when interpreting the causal effect in MR analysis [9, 37].

Finally, when interpreting these finding, several potential limitations of this MR meta-analysis should be considered. The first limitation was the retrieval of only English-language articles, which might result in a selection bias. However, this bias is unlikely to affect the validity of our findings because our Egger's tests and filled funnel plots suggested a low probability of publication bias. The second limitation might be inadequate study power in view of limited qualified studies and small sample sizes involved and this limitation became more obvious in some subgroup analyses. The third limitation was that although statistical heterogeneity was improved in some subgroups, other sources of clinical heterogeneity remained to be resolved. The fourth limitation lied in the incapability to justify some prerequisites of the MR method due to the unavailability of original data from each qualified study. The fifth limitation was that only one promoter polymorphism in *TGFB1* gene was selected as an instrument and it is encouraging to see whether this polymorphism in combination with another functional locus will enhance risk prediction for hepatocellular carcinoma.

In conclusion, our findings provide evidence for the significant association of *TGFB1* gene C-509T polymorphism with hepatocellular carcinoma in population-based studies and circulating TGF-β1 changes, and importantly enhanced circulating TGF-β1 was suggested to be causally associated with an increased risk of hepatocellular carcinoma by choosing *TGFB1* gene C-509T polymorphism as an instrument, the association warranting further validation in large, independent studies. Considering the ubiquity of genetic heterogeneity and in view of sample sizes involved, our findings should be viewed to be preliminary until further confirmation in future larger, well-designed studies.

## MATERIALS AND METHODS

### Literature search

The Medline (http://www.ncbi.nlm.nih.gov/pubmed/) and Embase (http://store.elsevier.com/embase) were searched to identify articles that examined the association of *TGFB1* gene C-509T polymorphism with hepatocellular carcinoma and/or TGF-β1 changes in circulation before July 2016. Key terms included (“hepatocellular carcinoma” or “hepatocellular cancer” or “liver cancer” in title) and (“transforming growth factor beta” or “TGFB” or “TGFbeta” or “TGF-beta” in abstract) and (“genotype” or “allele” or “polymorphism” or “variant” or “mutation” in abstract). All retrieved articles were managed by the EndNote X7 software (Thomson Reuters EndNote). For major reviews, meta-analyses and original investigations, we also checked the bibliographies to avoid possible missing articles. The implementation of this meta-analysis abides by the guidelines in the Preferred Reporting Items for Systematic Reviews and Meta-analyses (PRISMA) statement [38].

### Inclusion criteria

Articles that examined the association of *TGFB1* gene C-509T polymorphism with hepatocellular carcinoma and/or TGF-β1 changes in circulation were included pending sufficient information to deduct odds ratio (OR) and weighted mean difference (WMD), as well as their 95% confidence interval (95% CI). Moreover, only articles published in English language were taken into account. Additionally, source of study participants must be described clearly in each study.

### Data collection

From each article, necessary data were collected and typed into a standard form separately by two authors (Wei-Qun LU and Ji-Liang QIU) and after cross-checking, disagreements were resolved by consensus. Necessary data incorporated the first author's surname name, year of publication, country, source of controls, genotyping platform, matching status, sample size, the genotype counts of *TGFB1* gene C-509T polymorphism or/and the mean and standard deviation values of circulating TGF-β1 across C-509T three genotypes and if available age, gender composition and the percentages of hepatitis B and C virus infection.

### Statistical analysis

The risk prediction of *TGFB1* gene C-509T polymorphism for hepatocellular carcinoma was measured by the ORs with 95% CIs in a random-effects model using the DerSimonian/Laird method [39]. The mean changes of circulating TGF-β1 across genotypes were measured by the WMDs with 95% CIs.

Statistical heterogeneity between studies was measured by inconsistency index (*I*^2^). The *I*^2^ represents the percentage of observed diversity resulting from heterogeneity rather than from chance. Higgins et al suggested an *I*^2^ of 50% or over as significant heterogeneity [40]. Besides statistical heterogeneity, clinical evidence of heterogeneity is also no less important and it is usually evaluated by subgroup analysis and meta-regression analysis. In this meta-analysis, subgroup analysis was performed according to country, source of controls, genotype method and sample size, respectively. Meta-regression analysis was performed by modeling age, gender and the percentages of hepatitis B and C virus infection individually.

It is widely accepted that any study with a significant and positive result tends to receive more favorable publication decisions than the equally well-conducted study with a nonsignificant and negative result, the phenomenon known as publication bias [41]. Several criteria have been developed to quantify the probability of publication bias and in this meta-analysis Egger's test and filled funnel plot were adopted to judge this bias. The significance level of Egger's test is set at 10%, a commonly held cutoff value.

On the premise of the significant association of *TGFB1* gene C-509T polymorphism with hepatocellular carcinoma and circulating TGF-β1 changes, the MR method is used accordingly to infer the cause-and-effect relationship. Under the principles of MR put forwarded by Katan MB in 1986 [42], the causal risk prediction of circulating TGF-β1 for hepatocellular carcinoma was calculated as the ratio of the coefficient of the association between *TGFB1* gene C-509T polymorphism and hepatocellular carcinoma to the ratio of the coefficient of the association between this polymorphism and circulating TGF-β1 changes. In detail, suppose that OR_TT-vs-CC_ is the risk estimate of the -509TT genotype vis-à-vis the -509CC genotype for hepatocellular carcinoma and ΔP is the mean difference in circulating TGF-β1 between the -509TT genotype and the -509CC genotype, and then the causal estimate of circulating TGF-β1 for hepatocellular carcinoma is calculated as OR_TT-vs-CC_^k/ΔP^ for per k change in TGF-β1 [43].

All statistical calculations aforementioned were done with Stata software version 12.0 for Windows (StataCorp, College Station, Texas, USA).

## References

[1] Sitaram RT, Mallikarjuna P, Landstrom M, Ljungberg B (2016). Transforming growth factor-beta promotes aggressiveness and invasion of clear cell renal cell carcinoma. Oncotarget.

[2] Kang HG, Chae MH, Park JM, Kim EJ, Park JH, Kam S, Cha SI, Kim CH, Park RW, Park SH, Kim YL, Kim IS, Jung TH (2006). Polymorphisms in TGF-beta1 gene and the risk of lung cancer. Lung Cancer.

[3] Yue D, Zhang Z, Li J, Chen X, Ping Y, Liu S, Shi X, Li L, Wang L, Huang L, Zhang B, Sun Y, Zhang Y (2015). Transforming growth factor-beta1 promotes the migration and invasion of sphere-forming stem-like cell subpopulations in esophageal cancer. Exp Cell Res.

[4] Frei K, Gramatzki D, Tritschler I, Schroeder JJ, Espinoza L, Rushing EJ, Weller M (2015). Transforming growth factor-beta pathway activity in glioblastoma. Oncotarget.

[5] Tas F, Yasasever CT, Karabulut S, Tastekin D, Duranyildiz D (2015). Serum transforming growth factor-beta1 levels may have predictive and prognostic roles in patients with gastric cancer. Tumour Biol.

[6] Li J, Mu S, Mu L, Zhang X, Pang R, Gao S (2015). Transforming growth factor-beta-1 is a serum biomarker of radiation-induced pneumonitis in esophageal cancer patients treated with thoracic radiotherapy: preliminary results of a prospective study. Onco Targets Ther.

[7] Radwan MI, Pasha HF, Mohamed RH, Hussien HI, El-Khshab MN (2012). Influence of transforming growth factor-beta1 and tumor necrosis factor-alpha genes polymorphisms on the development of cirrhosis and hepatocellular carcinoma in chronic hepatitis C patients. Cytokine.

[8] Neuzillet C, de Gramont A, Tijeras-Raballand A, de Mestier L, Cros J, Faivre S, Raymond E (2014). Perspectives of TGF-beta inhibition in pancreatic and hepatocellular carcinomas. Oncotarget.

[9] Smith GD, Ebrahim S (2003). ‘Mendelian randomization’: can genetic epidemiology contribute to understanding environmental determinants of disease?. Int J Epidemiol.

[10] Burgess S, Butterworth A, Malarstig A, Thompson SG (2012). Use of Mendelian randomisation to assess potential benefit of clinical intervention. BMJ.

[11] Niu W, Gu M (2016). Adding Mendelian randomization to a meta-analysis-a burgeoning opportunity. Tumour Biol.

[12] Walsh KM, Codd V, Rice T, Nelson CP, Smirnov IV, McCoy LS, Hansen HM, Elhauge E, Ojha J, Francis SS, Madsen NR, Bracci PM, Pico AR (2015). Longer genotypically-estimated leukocyte telomere length is associated with increased adult glioma risk. Oncotarget.

[13] Jiang Y, Chen X, Tian W, Yin X, Wang J, Yang H (2014). The role of TGF-beta1-miR-21-ROS pathway in bystander responses induced by irradiated non-small-cell lung cancer cells. Br J Cancer.

[14] Miao ZF, Zhao TT, Wang ZN, Miao F, Xu YY, Mao XY, Gao J, Wu JH, Liu XY, You Y, Xu H, Xu HM (2014). Transforming growth factor-beta1 signaling blockade attenuates gastric cancer cell-induced peritoneal mesothelial cell fibrosis and alleviates peritoneal dissemination both *in vitro* and *in vivo*. Tumour Biol.

[15] Romano G, Santi L, Bianco MR, Giuffre MR, Pettinato M, Bugarin C, Garanzini C, Savarese L, Leoni S, Cerrito MG, Leone BE, Gaipa G, Grassilli E (2016). The TGF-beta pathway is activated by 5-fluorouracil treatment in drug resistant colorectal carcinoma cells. Oncotarget.

[16] Ma J, Liu YC, Fang Y, Cao Y, Liu ZL (2015). TGF-beta1 polymorphism 509C > T is associated with an increased risk for hepatocellular carcinoma in HCV-infected patients. Genet Mol Res.

[17] Qi P, Chen YM, Wang H, Fang M, Ji Q, Zhao YP, Sun XJ, Liu Y, Gao CF (2009). −509C > T polymorphism in the TGF-beta1 gene promoter, impact on the hepatocellular carcinoma risk in Chinese patients with chronic hepatitis B virus infection. Cancer Immunol Immunother.

[18] Shah R, Hurley CK, Posch PE (2006). A molecular mechanism for the differential regulation of TGF-beta1 expression due to the common SNP -509C-T (c. -1347C > T). Hum Genet.

[19] Kim YJ, Lee HS, Im JP, Min BH, Kim HD, Jeong JB, Yoon JH, Kim CY, Kim MS, Kim JY, Jung JH, Kim LH, Park BL (2003). Association of transforming growth factor-beta1 gene polymorphisms with a hepatocellular carcinoma risk in patients with chronic hepatitis B virus infection. Exp Mol Med.

[20] Falleti E, Fabris C, Toniutto P, Fontanini E, Cussigh A, Bitetto D, Fornasiere E, Avellini C, Minisini R, Pirisi M (2008). TGF-beta1 genotypes in cirrhosis: relationship with the occurrence of liver cancer. Cytokine.

[21] Miki D, Ochi H, Hayes CN, Abe H, Yoshima T, Aikata H, Ikeda K, Kumada H, Toyota J, Morizono T, Tsunoda T, Kubo M, Nakamura Y (2011). Variation in the DEPDC5 locus is associated with progression to hepatocellular carcinoma in chronic hepatitis C virus carriers. Nat Genet.

[22] Shi HZ, Ren P, Lu QJ, Niedrgethmnn M, Wu GY (2012). Association between EGF, TGF-beta1 and TNF-alpha gene polymorphisms and hepatocellular carcinoma. Asian Pac J Cancer Prev.

[23] Xin Z, Zhang W, Xu A, Zhang L, Yan T, Li Z, Wu X, Zhu X, Ma J, Li K, Li H, Liu Y (2012). Polymorphisms in the potential functional regions of the TGF-beta 1 and TGF-beta receptor genes and disease susceptibility in HBV-related hepatocellular carcinoma patients. Mol Carcinog.

[24] Yang Y, Qiu XQ, Yu HP, Zeng XY, Bei CH (2012). TNF-alpha -863 polymorphisms and the risk of hepatocellular carcinoma. Exp Ther Med.

[25] Wan PQ, Wu JZ, Huang LY, Wu JL, Wei YH, Ning QY (2015). TGF-beta1 polymorphisms and familial aggregation of liver cancer in Guangxi, China. Genet Mol Res.

[26] Torre LA, Bray F, Siegel RL, Ferlay J, Lortet-Tieulent J, Jemal A (2015). Global cancer statistics, 2012. CA Cancer J Clin.

[27] Yu L, Cheng YJ, Cheng ML, Yao YM, Zhang Q, Zhao XK, Liu HJ, Hu YX, Mu M, Wang B, Yang GZ, Zhu LL, Zhang S (2015). Quantitative assessment of common genetic variations in HLA-DP with hepatitis B virus infection, clearance and hepatocellular carcinoma development. Sci Rep.

[28] Wang P, Li H, Shi B, Que W, Wang C, Fan J, Peng Z, Zhong L (2016). Prognostic factors in patients with recurrent hepatocellular carcinoma treated with salvage liver transplantation: a single-center study. Oncotarget.

[29] Xiang TX, Cheng N, Li XN, Wu XP (2012). Association between transforming growth factor-beta1 polymorphisms and hepatocellular cancer risk: A meta-analysis. Hepatol Res.

[30] Guo Y, Zang C, Li Y, Yuan L, Liu Q, Zhang L, Li S (2013). Association between TGF-beta1 polymorphisms and hepatocellular carcinoma risk: a meta-analysis. Genet Test Mol Biomarkers.

[31] Giannelli G, Villa E, Lahn M (2014). Transforming growth factor-beta as a therapeutic target in hepatocellular carcinoma. Cancer Res.

[32] Bierie B, Moses HL (2006). Tumour microenvironment: TGFbeta: the molecular Jekyll and Hyde of cancer. Nat Rev Cancer.

[33] Dong ZZ, Yao DF, Yao M, Qiu LW, Zong L, Wu W, Wu XH, Yao DB, Meng XY (2008). Clinical impact of plasma TGF-beta1 and circulating TGF-beta1 mRNA in diagnosis of hepatocellular carcinoma. Hepatobiliary Pancreat Dis Int.

[34] Ji F, Fu SJ, Shen SL, Zhang LJ, Cao QH, Li SQ, Peng BG, Liang LJ, Hua YP (2015). The prognostic value of combined TGF-beta1 and ELF in hepatocellular carcinoma. BMC Cancer.

[35] Dhanasekaran R, Nakamura I, Hu C, Chen G, Oseini AM, Seven ES, Miamen AG, Moser CD, Zhou W, van Kuppevelt TH, van Deursen JM, Mounajjed T, Fernandez-Zapico ME (2015). Activation of the transforming growth factor-beta/SMAD transcriptional pathway underlies a novel tumor-promoting role of sulfatase 1 in hepatocellular carcinoma. Hepatology.

[36] Sheehan NA, Didelez V, Burton PR, Tobin MD (2008). Mendelian randomisation and causal inference in observational epidemiology. PLoS Med.

[37] Smith GD, Ebrahim S (2004). Mendelian randomization: prospects, potentials, and limitations. Int J Epidemiol.

[38] Moher D, Liberati A, Tetzlaff J, Altman DG (2009). Preferred reporting items for systematic reviews and meta-analyses: the PRISMA statement. BMJ.

[39] DerSimonian R, Laird N (1986). Meta-analysis in clinical trials. Control Clin Trials.

[40] Higgins JP, Thompson SG, Deeks JJ, Altman DG (2003). Measuring inconsistency in meta-analyses. BMJ.

[41] Sridharan L, Greenland P (2009). Editorial policies and publication bias: the importance of negative studies. Arch Intern Med.

[42] Katan MB (1986). Apolipoprotein E isoforms, serum cholesterol, and cancer. Lancet.

[43] Minelli C, Thompson JR, Tobin MD, Abrams KR (2004). An integrated approach to the meta-analysis of genetic association studies using Mendelian randomization. Am J Epidemiol.

